# Self-Reported Alcohol Use and Abuse by Arrestees in the 1998 Arrestee Drug Abuse Monitoring Program

**Published:** 2001

**Authors:** Susan E. Martin, Kendall Bryant, Nora Fitzgerald

**Affiliations:** Susan E. Martin, Ph.D, and Kendall Bryant, Ph.D., are health scientist administrators at the National Institute on Alcohol Abuse and Alcoholism, Bethesda, Maryland. Nora Fitzgerald is a social science analyst at the National Institute of Justice, Washington, D.C

**Keywords:** AODR (alcohol or other drug [AOD] related) crime, arrest, offender, AODR violence, self-report, AOD abuse, demographic characteristics, gender differences, ethnic differences, age differences

## Abstract

Data collected in the Arrestee Drug Abuse Monitoring (ADAM) program on alcohol and other drug use among arrestees provide a valuable opportunity to examine the relationship between alcohol use and violence. The data are used to explore the combined use of alcohol and other drugs among offenders and the relationships between substance use and the offenders’ demographic characteristics and offenses. These findings are used to identify changes in the offenders’ alcohol and other drug use over time.

Although alcohol is legally available to adults age 21 and older and is the most widely used drug in the United States, the relationship between alcohol use and crime is the focus of less criminal justice research and receives less public attention compared with the research and attention devoted to the links between illicit drug use and crime. This article examines data collected from arrestees concerning their alcohol and other drug use prior to their offenses and explores the relationships among alcohol use, illicit drug use, arrestee characteristics, and charges filed at arrest as well as the implications of these findings.

Since the mid-1980s, the National Institute of Justice (NIJ) has tracked drug use by the persons arrested (i.e., detained by police) and booked (i.e., taken to central police facility for a preliminary judicial hearing) in the United States through its Drug Use Forecasting (DUF) system. In 1995 the survey instrument was modified and expanded. In 1998 the system was renamed the Arrestee Drug Abuse Monitoring (ADAM) program and increased from 24 to 35 cities with improved sampling procedures. Although the ADAM program continues to focus primarily on illicit drug use—including types of drugs used by arrestees, self-reported dependence, and the relationship between drug use and certain types of offenses— self-reported alcohol-use information is also collected. Such data allow researchers to examine the association between recent alcohol consumption and intoxication (both alone and in combination with other drugs) and arrests for various types of crimes. This article explores those associations.

The ADAM data provide a unique perspective on the relationships between alcohol and other drug use and crime. Most other studies of alcohol and crime rely on the victims’ reports of offenders’ drinking (e.g., the National Crime Victimization Survey [NCVS], conducted annually by the Bureau of Justice Statistics [BJS]); self-report surveys of correctional populations or periodic BJS censuses of Federal, State, and local correctional facilities; arrest data from the Federal Bureau of Investigation’s Uniform Crime Reporting program; archival data from arrest records in specific jurisdictions; or select data on specific crimes.

Studies based on victimization data may understate alcohol’s presence, particularly in cases where the offender was a stranger, because the victim is less likely to have observed the offender drinking or to have known his or her drinking status. Studies based on correctional populations’ self-reports tend to suffer from problems with retrospective recall that may either underestimate or overestimate alcohol’s presence^1^ and, subsequently, form a sample skewed toward the most serious offenders (i.e., people who have been convicted of crimes for which they have been incarcerated). Studies that rely on archival arrest report data also may understate alcohol’s presence, because the studies’ accuracy depend on how accurately police monitor and report the information, a process which is not consistent. Conversely, ADAM data are gathered from a representative sample of people who have been arrested for a wide spectrum of offenses and the arrestees’ reports on their alcohol or other drug use shortly after the substance-use occurs. In addition, the arrestees’ urine is tested for the presence of illicit drugs.

## Methods

Arrestees who participate in the ADAM program are surveyed concerning their substance use and, at the conclusion of the interview, are asked to provide a urine specimen, which is screened for the presence of 10 illicit drugs. In 1998 data were obtained from 20,715 adult male arrestees who were interviewed at 35 sites and 6,699 adult female arrestees who were interviewed at 32 sites. The data collection area for each site included the county within which the city was located.^2^

For each quarter in 1998, trained local staff at each site obtained voluntary and anonymous interview data and urine specimens from detained arrestees who had been in the booking facility for not more than 48 hours. On average, each site sought to obtain a sample of 225 males and 100 females per quarter. All arrestees booked at a facility within the previous 48 hours were eligible for interviews. However, because more male arrestees were available than could be interviewed, the sample of men was selected on the designated interview days using the following order of priority based on reason for arrest: (1) nondrug felony, (2) nondrug misdemeanor, (3) drug felony, and (4) drug misdemeanor. Sites were asked to survey no more than 20 percent of the males charged with drug offenses. All women arrestees were eligible for an interview regardless of their charges.

Approximately 80 percent of eligible arrestees agreed to be interviewed. Of those who agreed to the interview, about 80 percent also provided a urine specimen. Only persons who agreed to the interview and provided a specimen were included in the data (i.e., nearly two-thirds of eligible arrestees). (For further information about data collection procedures and the data set, see the U.S. Department of Justice 1999.)

During the ADAM interview, arrestees were asked about their use of both legal and illegal drugs, including alcohol. Alcohol-related questions addressed the interviewed arrestees’ lifetime, past year, and past month alcohol use as well as alcohol use within the previous 72 hours (termed “recent alcohol use” throughout this article). Interviewers also asked arrestees if they were “under the influence” of alcohol at the time of the offense for which they were arrested. Similar questions were asked about each of a number of illicit drugs.

We analyzed two alcohol-related ADAM items: (1) recent alcohol use and (2) being “under the influence of alcohol” at the time of the offense (also termed alcohol abuse or intoxication). In this article, we present findings for both measures in order to provide a more complete picture of the role of alcohol in crime.

The ADAM survey instrument does not include questions related to quantity of alcohol consumed. Because the arrestees who reported recent alcohol use may have consumed an unspecified amount of alcohol and done so at any point during the 72 hours before being arrested, one cannot infer from this question that alcohol use affected the arrestee’s behavior. Asking arrestees whether they were “under the influence” yields a subjective self-report measure that may be systematically biased. In contrast to the self-reported alcohol measures, the illicit drug data are based on positive urinalysis. However, neither urinalysis nor breath testing for alcohol use is practical as part of the ADAM data collection, because alcohol is metabolized so quickly.

To address threats to the validity of the data that may be associated with self-reporting alcohol use, we employed three strategies. First we examined self-reports of persons whose responses to drug-use questions were inconsistent with their urine specimens and compared them with consistent reporters to determine if their levels of alcohol use differed. Second, we compared ADAM self-reported alcohol use and abuse data by age group with similar data from a nationally representative survey of alcohol and other drug use. Third, we reported both alcohol use and abuse, based on the assumption that recent use (i.e., drinking any alcohol in the 72 hours before the arrest) overstated the possible effect of drinking on criminal behavior, whereas self-reported “being under the influence” at the time of the arrest probably understated alcohol’s effects.

We examined the ADAM data to uncover subjects with discrepancies between urine test findings and self-reported use of marijuana, crack, and cocaine and labeled them as “inconsistent.” We then compared consistent and inconsistent arrestees regarding several measures of drug use. Using two cocaine-related variables, respectively, we identified (1) persons who admitted having used crack or cocaine but denied using it in the past 30 days although their urine tested positive (5.8 percent of the total sample) and (2) persons who denied ever using crack or cocaine in the previous month but whose urine tested positive (an additional 8 percent of the sample).^3^ Similarly, two marijuana consistency measures identified (1) arrestees who denied use in the past month, admitted to having tried marijuana in the past, but tested positive (4.5 percent of the total) and (2) arrestees who denied ever using marijuana but tested positive for the drug (an additional 1.2 percent of the total). Three additional variables were created to identify arrestees who admitted ever using any of the aforementioned drugs but denied recent use (9.9 percent); arrestees who denied ever using either marijuana, crack, or cocaine but tested positive (9.5 percent of the sample); and arrestees who gave an inconsistent report on any of the validity checks (18.7 percent of the sample).

Like Harrison (1995) who studied DUF arrestees, we found reasonable congruence between self-report and urinalysis among respondents and drug-use underreporting based on social desirability. Thus, marijuana use was more likely to be reported than cocaine use, and ever use was more likely to be reported than recent use.

To assess how much arrestees underreported their alcohol use and abuse, we compared the self-reports of alcohol use and abuse for the respondents who gave consistent self-reports and urine with the inconsistent reporters. In our ADAM sample, no statistical difference existed in the percentage of respondents who reported recent alcohol use among those who had inconsistent self-reports and urine tests regarding recent or lifetime use of marijuana, cocaine, or crack and respondents whose drug tests and self-reports were congruent. With respect to being under the influence at the time of the offense, no difference existed between respondents who gave self-reports about recent drug use inconsistent with their urine tests and those who did not. However, respondents who denied ever using marijuana, cocaine, or crack but who tested positive for one of those drugs were significantly less likely to report being under the influence of alcohol (16.6 percent) than were the consistent reporters (21.1 percent). Because the number of persons in this group was quite small, however, we did not adjust the data or delete this group from the data reported here.^4^

To address possible greater underreporting of alcohol consumption by underage offenders than those persons legally permitted to drink, we compared ADAM data on drinking during the previous 30 days, broken down by age group, with similar data from the 1998 National Household Survey of Drug Abuse (NHSDA) (SAMHSA 1999). Although the percentage of respondents who reported using alcohol in the past month was consistently higher among ADAM arrestees than the household survey respondents for each age category, the age-related patterns of use for both samples were similar. For example, in the NHSDA for 1998, 32 percent of 16- and 17-year-olds, 54 percent of 18- to 20-year-olds, 65 percent of 21- to 25-year-olds, and 53 percent of people age 35 and older reported drinking in the past month. Among the ADAM arrestee sample, the comparable figures are 54 percent for 16- and 17-year-olds, 67 percent for 18- to 20-year-olds, 73 percent for 21- to 25-year-olds, and more than 70 percent for arrestees older than 25. Thus the lower rate of self-reported alcohol use among persons less than age 21 is consistent with national data and is not likely to be caused by reporting bias.

## Findings

The ADAM data provide an opportunity to explore the combination of alcohol use, drug presence, and offense data among recent arrestees; to examine possible links between alcohol use and abuse—alone and in combination with other drugs—and specific types of criminal behavior; and to identify changes in these associations since 1989, when the only DUF report containing alcohol data was published (Visher 1990). Given the ample literature indicating an association between alcohol use and violence (Murdoch et al. 1990; Parker and Rehbun 1995; Pernanen 1991; Roizen 1993; Lipsey et al. 1997), we focused specifically on the presence and influence of alcohol in violent crimes compared with other offenses, particularly property crimes.

### A View of the Arrestees

The sociodemographic characteristics of the 1998 ADAM arrestees are shown in table 1. The arrestees tended to be young (i.e., median age of 30), but ranged in age from 15 to 82. Most arrestees were male (75.6 percent), African-American (45.6 percent), had limited education (48 percent had not finished high school), and were employed either full time (42.5 percent) or part time (18 percent) during the month prior to their arrests.

The vast majority of both male and female 1998 arrestees reported that they tried alcohol at some time during their lives (94 and 89 percent, respectively), and most had also consumed alcohol during the past year (80 and 70 percent, respectively). As shown in table 2, more than one-half of all male arrestees (54 percent), but somewhat less than one-half of the female arrestees (43 percent), reported drinking in the 72 hours prior to their arrests, and 22 percent of the males and 16 percent of the females reported being under the influence of alcohol at the time of their offenses. The gender difference in arrestees’ reported drinking and drunkenness reflects societal alcohol consumption patterns whereby men drink greater quantities and more frequently than women (SAMHSA 1999).^5^

**Figure 1:**
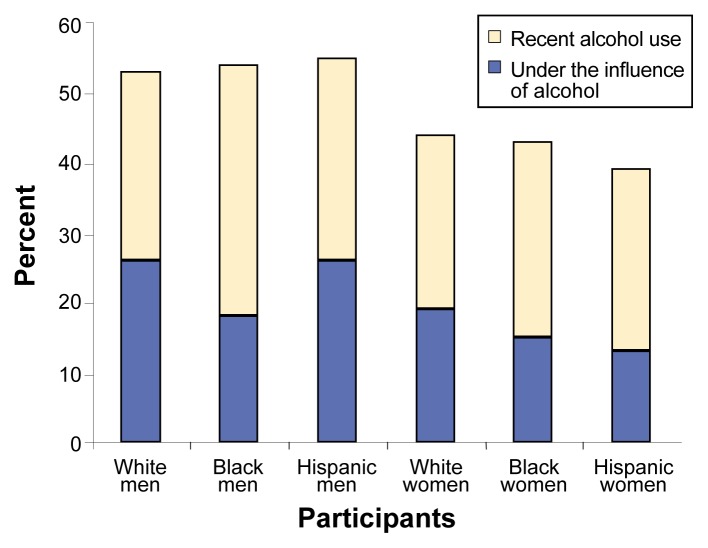
Alcohol use and abuse among 1998 ADAM system participants by race/ethnicity and gender. Virtually no racial differences were found in recent alcohol consumption among white, black, and Hispanic male arrestees. A smaller proportion of black men than white or Hispanic men reported being under the influence at the time of the offense. Among the women, Hispanics were slightly less likely than whites or blacks to report recent alcohol use and less likely to have been under the influence at the time of the offense. ADAM = Arrestee Drug Abuse Monitoring. SOURCE: U.S. Department of Justice 1999.

Virtually no racial differences were found in recent alcohol consumption among white, black, and Hispanic male arrestees. However, as shown in figure 1, a smaller proportion of black men (18 percent) than either white men (26 percent) or Hispanic men (26 percent) reported being under the influence at the time of their offenses. Among the women, Hispanics were less likely than either whites or blacks to report recent alcohol use (39 percent versus 44 and 43 percent, respectively) and less likely to have been under the influence at the time of their offenses (13 percent versus 19 and 15 percent, respectively). Together these results indicate that virtually no difference was found between black men and black women in the likelihood of being under the influence of alcohol at the time of the offense, whereas Hispanic men were twice as likely as were Hispanic women to have been under the influence of alcohol.

**Figure 2:**
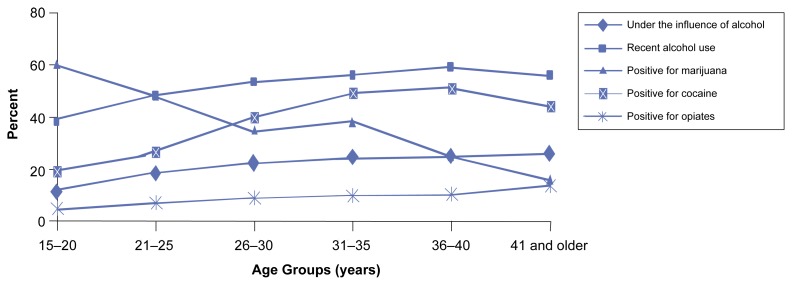
Alcohol and other drug use/abuse by age group. In all age groups except persons under age 21, arrestees were more likely to report recent alcohol use than test positive for cocaine, marijuana, or opiates. Arrestees between ages 15 and 20 were more likely to test positive for marijuana than to have reported either recent alcohol use or being under the influence of alcohol at the time of their offense. SOURCE: U.S. Department of Justice 1999.

Use and abuse of alcohol also varied by the age of the arrestee. In all age groups except persons under age 20, arrestees were more likely to report recent alcohol use than to test positive for cocaine, marijuana, or opiates. As shown in figure 2, arrestees between ages 15 and 20 were more likely to test positive for marijuana (59 percent) than to have reported either recent alcohol use (38 percent) or being under the influence of alcohol (11 percent) at the time of their offense. The high rate of marijuana use among young arrestees reflects the recent rise reported in the Monitoring the Future (MTF) survey (Johnston et al. 1999), which tracks a nationally representative sample of young adults. The rate of marijuana use among arrestees, however, is much higher than that of the MTF sample of persons of the same age.^6^

Although the rates of alcohol use and abuse among arrestees remain steady with increasing age, other drug use gradually declines. Use of marijuana rapidly drops off after age 21; cocaine use rises through the 20s, peaks in the 30s, but drops after age 40. In contrast, rates of alcohol use and abuse also rise rapidly during the 20s, but remain virtually unchanged through the 40s; just over one-fourth of the arrestees age 35 and older were under the influence of alcohol at the time of their offense.

Neither drinking nor intoxication differed by arrestees’ level of education, but both were higher among those who were working full time than were those with other sources of support. For example, 23 percent of full-time workers and 19 percent of respondents who had other sources of income were under the influence at the time of their offense.

### Charge at Arrest, Gender, and Alcohol Use and Abuse

The ADAM findings reveal important differences in the proportion of arrestees who reported recent alcohol use and abuse by their charge at arrest and their gender. As shown in table 2, men were more likely than were women to have been drinking recently before their arrest for every type of offense. As would be expected, men and women arrested for driving under the influence (DUI) or driving while intoxicated (DWI) were more likely than arrestees charged with any other offense to report recent drinking, followed by arrestees for public disorder offenses. Recent drinking was reported by 71 percent of the women and 80 percent of the men arrested for DUI/DWI and by 61 and 74 percent, respectively, of women and men arrested for public disorder offenses. Men were much more likely than were women to report recent drinking prior to family offenses (57 versus 42 percent, respectively) and homicides (52 versus 37 percent).

Similarly, male arrestees were more likely than female arrestees to report being under the influence of alcohol at the time of the offense for most types of offenses except for homicide, where the numbers become quite small.^7^ In addition, little difference occurred in the proportion of men and women who reported being under the influence when arrested for DWI and for “other violence” (including the serious but infrequent offenses of kidnapping, manslaughter, and blackmail or threats), and modest differences existed in the proportions of men and women arrested for assaults and family violence who reported being under the influence.

Both men and women were about as likely to have used alcohol prior to arrest for a violent crime as for a property or drug offense. However, the proportion of drug and property offenders of both genders who were under the influence of alcohol at the time of their offense is substantially smaller than the proportion of violent offenders who were intoxicated. The strong association between alcohol intoxication and violent crimes compared with other types of crimes by both men and women is similar to the findings of many other studies (Wolfgang 1958; Murdoch et al. 1990; Parker and Rehbun 1995; Pernanen 1991; Roizen 1993; Lipsey et al. 1997). Research suggests that in about one-half of the violent incidents involving alcohol, one might attribute the violent outcome to the alcohol (Room and Rossow 2000). For additional information on the proportion of violent crime that is alcohol related, see the article in this issue by Greenfeld and Henneberg, pp. 20–31. In contrast to the patterns of alcohol use and abuse among arrestees, female arrestees were more likely than male arrestees to have used cocaine recently prior to their crime (44 percent versus 35 percent), particularly in the case of drug offenses (62 and 49 percent, respectively) (see table 2). In addition, arrestees who tested positive for cocaine tended to be apprehended for property offenses rather than for violent crimes. For example, 18 percent of the men and 10 percent of the women who tested positive for cocaine were arrested for a violent crime, whereas 25 percent of the men and 21 percent of the women who tested positive for cocaine were arrested for property offenses (data not shown). These findings support Visher’s (1990) observation that compared with alcohol use, “cocaine use is more closely tied to income-generating crime.”

### Alcohol and Cocaine Use Among Male Arrestees: 1989 and 1998

Compared with the 1989 DUF data reported by Visher (1990) on male arrestees, the rate of recent alcohol use among men was reduced among the 1998 ADAM participants, as was the proportion of men who tested positive for cocaine (no data were reported comparable to the “under the influence” measure). The decrease in cocaine use was substantially larger than the decrease in alcohol use. In 1989, 59 percent of the male arrestees reported recent use of alcohol and 50 percent tested positive for cocaine. In 1998 those figures were 54 and 35 percent, respectively. Nevertheless, men arrested for property offenses in 1998 continued to be more likely than those arrested for violent offenses to test positive for cocaine (40 versus 26 percent, respectively). In contrast, men arrested for violent offenses were more likely to report recent alcohol use than to test positive for cocaine (55 percent and 51 percent, respectively).

### Combined Alcohol and Drug Use Among Arrestees

Many arrestees were found to have used a combination of alcohol and other drugs before their arrest. Thirty-seven percent of the arrestees included in the 1998 ADAM data tested positive for 1 of 10 illegal substances and also reported recent use of alcohol. This finding includes 23 percent of arrestees who had recently consumed alcohol and 14 percent who reported being under the influence of alcohol at the time of their arrests and who also tested positive for an illicit drug. To further explore the combined use of alcohol and illicit drugs among arrestees, we examined data on six alcohol- and other drug-use combinations and analyzed the data by type of arrest charge. The categories of alcohol- and other drug-use combinations included the following: (1) no alcohol or other drug use, (2) recent alcohol use only (but not under the influence of alcohol), (3) under the influence of alcohol only, (4) under the influence of alcohol and positive for illicit drugs, (5) recent alcohol use and positive for illicit drugs, and (6) only positive for illicit drugs. Within these categories, we examined the percentage of persons charged with each of four broad offense groups: (1) violent crimes, (2) property crimes, (3) alcohol-related crimes (e.g., DUI), and (4) drug possession or sale.^8^

**Figure 3:**
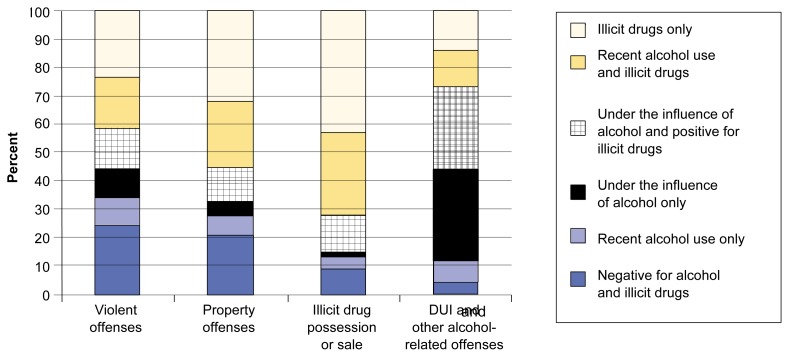
Alcohol- and drug-use combinations by type of charge at arrest. DUI = driving under the influence. SOURCE: U.S. Department of Justice 1999.

Among the arrestees charged with violent offenses, starting at the bottom of the left-most column in figure 3, a total of 24 percent reported no recent alcohol use and tested negative for illicit drugs; 10 percent had recently consumed alcohol and another 10 percent were intoxicated by alcohol but tested negative for drugs; 15 percent were under the influence of alcohol and positive for other drugs; 18 percent were drug positive and had recently consumed alcohol but were not intoxicated; and 23 percent had used illicit drugs only. In contrast, figure 3 shows that among arrestees charged with property offenses, a larger proportion were using only illicit drugs (32 percent); a smaller proportion had used no drugs or alcohol (21 percent), had recently consumed alcohol (7 percent), or were either intoxicated and negative for other drugs (5 percent) or intoxicated and positive for other drugs (12 percent); whereas nearly one-fourth of the property offenders reported recent alcohol and other drug use (24 percent). Twenty-six percent of violent offenders, but only 16 percent of property offenders, were self-reportedly under the influence of alcohol either with or without the presence of other drugs.

Figure 3 also indicates that 30 percent of those arrested for DUI and other alcohol-related offenses were negative for other drugs, and 27 percent were under the influence of alcohol and positive for other drugs. Conversely, persons arrested for the sale or possession of illicit drugs were most frequently using only illicit drugs (43 percent) or were positive for illicit drugs and reported recent alcohol use (29 percent). Arrestees in this group were the least likely to report only recent alcohol use or being under the influence of alcohol.

Comparison with the 1989 DUF data reported by Visher (1990) on combined alcohol and other drug use among arrestees showed that several notable changes in substance use occurred by 1998. Compared with the 1989 arrestees, a larger proportion of arrestees in 1998 were negative for both alcohol and illicit drugs. In addition, a larger proportion of the 1998 arrestees had used only illicit drugs, and a smaller proportion had used only alcohol. For example, the proportion of male property offenders who had used only illicit drugs decreased from 42 to 30 percent and the proportion of those using alcohol and cocaine decreased from 39 to 24 percent. The proportion of men arrested for violent offenses who had used only alcohol, however, increased from 28 percent to 33 percent.

## Discussion

This examination of alcohol and other drug use among arrestees who participated in the 1998 ADAM program has shown that both alcohol and illicit drug use are frequently associated with crime. One-half of the arrestees had recently consumed alcohol, and 21 percent were under its influence at the time of the crime, 37 percent tested positive for cocaine, and 29 percent tested positive for at least one other illicit drug (primarily marijuana). Men were more likely to have recently used alcohol or to be under its influence than were women, and female arrestees were more likely than were male arrestees to test positive for cocaine. As has been found in other studies, and contrary to the popular view that illicit drug use, rather than alcohol use, is associated with crime, the ADAM data suggest that except for robbery, alcohol intoxication is as likely as is cocaine use to precede violent crimes and is more likely than cocaine use to precede family violence. In contrast, cocaine use, and to a lesser extent other illicit drug use, is more likely than alcohol intoxication to be associated with property crime, although many property offenders had recently consumed alcohol. Although women are less likely to drink and less likely to commit violent crimes than men, the association between violent crime and alcohol intoxication is also observed among female arrestees and is most strongly linked with expressive offenses (i.e., those motivated primarily by emotion rather than by financial gain), including assaults and family violence.

Because the prevalence of alcohol use and abuse is quite high among arrestees, both alone and accompanied by illicit drug use, our findings suggest that to achieve further reductions in crime, particularly in violent crime, strategies must be implemented that focus on reducing alcohol abuse as well as illicit drug use. Such strategies might include both expanded programs to address the substance abuse treatment needs of individual offenders in the correctional system and strategies that address the environmental and situational factors that contribute to alcohol-related offenses. For example, policies such as limiting alcohol availability by raising the minimum legal drinking age and increasing the excise taxes on alcohol have been found to have had an unanticipated salutary effect on reducing crime (Cook and Moore 1993; Parker and Cartmill 1998).

The lack of information about the situational aspects of the offenses and limitations of the alcohol-related questions on the ADAM questionnaire necessitate a level of caution in interpreting our findings. A report of recent alcohol use is likely to overestimate the presence of alcohol effects at the time of the offense. Conversely, asking an arrestee whether he or she was under the influence of alcohol at the time of the crime probably leads to underestimates in the role of alcohol, given the general tendency to underreport the effects of drinking. Nevertheless, some arrestees may overreport intoxication, because it sometimes is seen as a mitigating factor in explaining behavior. In addition, whether someone is under the influence is inconsistently measured. This measure approximates an “attributable fraction” (i.e., the proportion of incidents that can be attributed to alcohol use) of instances in which alcohol may have had a casual role in the offense (Room and Rossow 2000). Despite these limitations, the ADAM data permit a useful examination of various alcohol-and other drug-use combinations and their relationships with specific types of crimes and lend support to other studies that highlight the role of alcohol use and abuse in violence, particularly in violent crimes. Further analyses might explore the association of alcohol with specific types of violent offenses, probe trends in the association of alcohol with these offenses, and consider various approaches to further weaken the alcohol-violence nexus.

## Figures and Tables

**Table 1:** Characteristics of 1998 ADAM* Program Arrestees

Characteristics	Number	Percent
Age		
Mean	31.1	NA
Median	30.0	NA
Range	15–82	NA
Gender		
Male	20,715	75.6
Female	6,699	24.4
Race/ethnicity		
Black (non-Hispanic)	12,460	45.6
White (non-Hispanic)	8,456	30.9
Hispanic	5,484	20.7
Other	737	2.9
Education		
Less than high school graduate	12,045	47.7
High school diploma or GED	8,372	33.3
Some college	4,706	18.7
Employment		
Working full time	11,589	42.5
Other sources of legal income	11,524	42.3
Illegal sources of income	2,359	8.7
No income	1,790	6.6
Arrest in the previous year	10,926	39.9

* ADAM = Arrestee Drug Abuse Monitoring.

The ADAM program is a system used by the National Institute of Justice to track drug use by a representative sample of people in the United States who have been arrested and charged for a wide variety of criminal offenses. NA = not applicable; GED = general equivalency diploma.

**Table 2:** Alcohol and Cocaine Use/Abuse Among Percentages of 1998 ADAM^1^ Arrestees by Gender and Charge

	Recent Alcohol Use^2^		Under the Influence^3^		Cocaine Use	
	
Arrest Charge	Female (*n* = 6,659) (%)	Male (*n* = 20,612) (%)	Female (*n* = 6,659) (%)	Male (*n* = 20,612) (%)	Female (*n* = 6,664) (%)	Male (*n* = 20,630) (%)
**Violent Offense**	44	55	23	26	26	26
Assault	47	57	24	29	28	29
Homicide	37	52	16	14	21	17
Robbery	39	54	13	21	51	39
Sexual assault	NA	52	NA	19	NA	16
Weapons	23	46	7	18	20	24
Family violence	42	57	26	30	18	20
Other violence	49	54	23	24	25	25
**Property-Related Offense**	36	51	14	18	40	40
Burglary	35	50	8	19	41	41
Theft	34	52	8	16	37	42
Auto theft	42	45	13	16	39	40
Forgery/fraud	29	41	5	10	24	28
Other property	49	62	18	30	64	41
**Other Offense**						
DUI/DWI	71	80	61	64	27	20
Drug sale/possession	43	49	12	15	62	49
Public disorder	61	74	22	39	43	31
Flight/warrant	38	53	10	18	37	32
Other misc. offenses	47	54	19	22	48	32

**Total**	**43**	**54**	**16**	**22**	**44**	**35**

1 ADAM = Arrestee Drug Abuse Monitoring.

The ADAM program is a system used by the National Institute of Justice to track drug use by a representative sample of people in the United States who have been arrested and charged for a wide variety of criminal offenses.

2 Recent alcohol use refers to alcohol use within the previous 72 hours.

3 Respondents are also asked whether they were “under the influence” of alcohol at the time of the offense for which they were arrested.

DUI/DWI = driving under the influence/driving while intoxicated; NA = not applicable.

## Footnotes

1 Although most studies find that underreporting increases when the length of the recall period is more than 1 year, a BJS survey of prisoners calculated their blood alcohol concentration (BAC) based on self-reported drinking at the time of the offense and estimated that men had an average BAC of 0.27 percent and women had an average BAC of 0.37 percent (Greenfeld 1998).

2 Two exceptions exist: (1) Anchorage data represent only that city, and (2) because New York City includes five separate counties, data are drawn from each of those boroughs.

3 Although cocaine can be detected in urine for approximately 36 hours, metabolites of cocaine can be detected for up to 2 or 3 weeks in heavy users. For that reason, as well as to be consistent in measures across drugs, we adopted a conservative consistency measure for recent cocaine use.

4 We estimate that the maximum number of persons who may have been dissimulating about alcohol intoxication was 107 respondents out of more than 27,000.

5 For example, 59 percent of the men but 45 percent of the women surveyed in the 1998 NHSDA reported drinking in the previous month, and 10 percent of the men and 2 percent of the women reported heavy drinking during that time (SAMHSA 1999).

6 For example, among ADAM arrestees, 59 percent from ages 18 to 20 self-reported having used marijuana in the past month. In contrast, the Monitoring the Future survey found that between 20 and 23 percent of 18- to 20-year-olds had used marijuana in the previous 30 days (Johnston et al. 1999).

7 We have not reported statistical tests here, because the probability of finding a numerically significant difference is strongly affected by sample size. With more than 27,000 subjects, virtually all tests were positive.

8 Because the “other” category included persons arrested for a wide variety of offenses, including trespassing, gambling, prostitution, and parole violations, this category is not shown nor discussed.
